# Multivessel Coronary Artery Disease and Subsequent Thrombolysis in Myocardial Infarction Flow Grade After Primary Percutaneous Coronary Intervention

**DOI:** 10.7759/cureus.8752

**Published:** 2020-06-21

**Authors:** Haris Majeed, Muhammad N Khan, Khalid Naseeb, Najia A Soomro, Saeed Alam, Shahid Ahmed, Usman Bhatti, Tahir Saghir

**Affiliations:** 1 Cardiology, National Institute of Cardiovascular Diseases, Karachi, PAK; 2 Interventional Cardiology, National Institute of Cardiovascular Diseases, Karachi, PAK; 3 Cardiology, Liaquat National Hospital, Karachi, PAK; 4 Adult Cardiology, National Institute of Cardiovascular Diseases, Karachi, PAK

**Keywords:** coronary artery disease, multivessel disease, primary pci, timi flow

## Abstract

Background

In underdeveloped countries, coronary artery disease (CAD) has developed into a serious health issue due to the high rates of risk factors such as obesity and smoking amongst the population. This study has been performed to find the rate of multivessel CAD (MVD) and subsequent thrombolysis in myocardial infarction (TIMI) flow grade III in patients undergoing primary percutaneous coronary intervention (PCI).

Methods

This transverse study was carried out involving 110 patients from the emergency department of the National Institute of Cardiovascular Diseases, Karachi, Pakistan, from August 2015 to March 2016. All patients were diagnosed as ST-segment elevation myocardial infarction (STEMI) and had gone through primary PCI. Pre-procedure angiographic findings regarding the number of vessels involved and post-procedure TIMI flow grade were assessed and analysed.

Results

The average age of the study sample was 56.3 ± 11.4 years. The proportion of male patients was 81.8% (n=90), and hypertension was the most prevalent risk factor followed by type II diabetes with a frequency of 67.3% (n=74) and 40.0% (n=44), respectively. Coronary angiography showed MVD in 50.0% (n=55) of the patients, of whom 34 patients had two-vessel disease, and the remaining 21 had three-vessel disease. Ninety percent (n=99) of the patients exhibited TIMI flow grade III after the procedure with no significant difference between patients with MVD and those with single-vessel disease with a rate of 87.3% (n=48/55) versus 92.7% (n=51/55, P=0.527), respectively.

Conclusion

Post-procedure TIMI flow grade III was accomplished in almost 90% of the subjects with or without MVD. It can be concluded that primary PCI has a significant role in the early restoration of myocardial blood flow following STEMI regardless of the vessels involved.

## Introduction

Globally, cardiovascular diseases (CVDs) claim the highest number of lives and significantly contribute to poor quality of life each year [1]. In 2017, CVDs claimed 17.8 million lives and 35.6 million years of life lost due to disabilities [2,3]. Nearly 80% of this global burden consists of low- and middle-income populations [4].

The populations of South Asian countries are at the highest risk of developing the CVD; therefore, they experience the highest rates of death across the world [5-7]. Due to the fast growth of cities in this part of the world, more people have moved to urban centers. This has resulted in a spike of coronary artery disease (CAD) occurrence, and therefore it becomes essential to study, analyse, and understand which internal and external factors cause a high risk of disease so that strategies to prevent the disease may be developed [5].

The high rates of CAD in the South Asian population have been linked to an ongoing epidemiological transition and a higher prevalence of cardiac risk factors, such as hypertension (HTN), obesity, sedentary lifestyle, smoking, and type II diabetes mellitus (DM), in the region [3,8,9].

According to multiple studies involving clinical trials and institutional registries, multivessel CAD (MVD) was documented in around half of the patients with acute myocardial infarction (AMI). CAD involving multiple vessels has been linked to increased chances of mortality and morbidity amongst patients suffering from the disease [10-12].

The thrombolysis in myocardial infarction (TIMI) flow grade assessment is the standard criterion for the evaluation of coronary reperfusion yield. TIMI flow grade II or III of the native artery after a primary percutaneous coronary intervention (PCI) is indicative of the procedure success. Post-procedure TIMI flow grade III was found to be linked with decreased chances of death and morbidity, lower enzyme peaks, and improved global outcomes, including regional left ventricular function [13].

This study has been performed to find the rate of MVD and subsequent TIMI flow grade III in patients who have undergone primary PCI.

## Materials and methods

This cross-sectional study included 110 patients aged 18 to 82 years visiting the emergency department of the National Institute of Cardiovascular Diseases, Karachi, Pakistan, from August 2015 to March 2016. Patients of either sex with acute ST-segment elevation myocardial infarction (STEMI) were included using a nonprobability consecutive sampling technique. Informed consent was obtained from all the patients. Patients with a prior history of myocardial infarction (MI) were excluded.

Data regarding demographics (gender, age, height, and weight) and clinical characteristics were recorded for all the patients, which included a history of DM, HTN, dyslipidemia, smoking status, and family history of CAD. Body mass index (BMI) was calculated; clinical characteristics were defined based on clinical history. Patients with a documented history of HTN on antihypertensive medication for at least six months were categorized as hypertensive. Similarly, patients on medical therapy for DM for at least six months were categorized as DM. Patients were interviewed for the history of premature CAD in immediate blood relatives and last documented lipid profile, and dyslipidemia and family history of CAD were categorized.

After the diagnosis of STEMI, all the patients were managed as per the institutional protocols. Experienced cardiologists performed the primary PCI procedure. Patients with significant (>70%) stenosis in more than one vessel were categorized as MVD. PCI was attempted for the culprit artery only, and post-procedure TIMI flow grade was recorded.

Data were analysed on IBM SPSS Statistics for Windows, Version 21.0 (IBM Corp, Armonk, NY). Age, height, weight, and BMI were presented as means ± standard deviations (SDs), and frequency and percentages were presented for categorical variables including gender, DM, HTN, smoking status, dyslipidemia, MVD, and post-procedure TIMI flow grade. Effect modifiers like age, BMI, gender, DM, HTN, smoking status, and dyslipidemia were controlled through stratification. Post-stratification, appropriate chi-square test or Fisher’s exact test was applied; a P value ≤0.05 was considered significant.

## Results

The average age of our study sample was 56.3 ± 11.4 years, with 52.7% (n=58) patients ≤55 years of age. The proportion of male patients was 81.8% (n=90), and HTN was the most prevalent risk factor followed by DM with a frequency of 67.3% (n=74) and 40.0% (n=44), respectively. The overall mean BMI of study subjects was 25.22 ± 2.71 kg/m^2^ (range, 18.4-36.782 kg/m^2^). Coronary angiography showed MVD in 50.0% (n=55) of the patients, of whom, 34 patients had two-vessel disease, and the remaining 21 had three-vessel disease. Table 1 presents the demographic information and clinical values by disease severity.

**Table 1:** Features of the demography and clinical values by severity of coronary artery diseases

	Total	Disease severity		P value
		Multivessel disease	Single-vessel disease	
Total	110	55	55	-
Gender				
Male	81.8% (90)	70.9% (39)	92.7% (51)	0.003
Female	18.2% (20)	29.1% (16)	7.3% (4)	
Age groups				
≤55 years	52.7% (58)	50.9% (28)	54.5% (30)	0.702
>55 years	47.3% (52)	49.1% (27)	45.5% (25)	
Body mass index (kg/m^2^)				
≤25	57.3% (63)	60% (33)	54.5% (30)	0.563
>25	42.7% (47)	40% (22)	45.5% (25)	
Diabetes mellitus				
Yes	40% (44)	47.3% (26)	32.7% (18)	0.119
No	60% (66)	52.7% (29)	67.3% (37)	
Hypertension				
Yes	67.3% (74)	72.7% (40)	61.8% (34)	0.223
No	32.7% (36)	27.3% (15)	38.2% (21)	
Smoking				
Yes	48.2% (53)	41.8% (23)	54.5% (30)	0.182
No	51.8% (57)	58.2% (32)	45.5% (25)	
Dyslipidemia				
Yes	31.8% (35)	52.7% (29)	10.9% (6)	<0.001
No	68.2% (75)	47.3% (26)	89.1% (49)	
Family history of coronary artery disease				
Yes	20.9% (23)	36.4% (20)	5.5% (3)	<0.001
No	79.1% (87)	63.6% (35)	94.5% (52)	

Ninety percent of the patients exhibited post-procedure TIMI flow grade III with no significant difference between patients with MVD and those with single-vessel disease (SVD) with a rate of 87.3% (n=48/55) versus 92.7% (n=51/55; P=0.527), respectively. Table 2 presents demographic and clinical values by the severity of the TIMI flow grade III after the procedure.

**Table 2:** Features of the demography and clinical values by severity of the TIMI flow grade III after the procedure TIMI = thrombolysis in myocardial infarction

	Post-procedure TIMI flow grade		P value
	III	0-II	
Total	90	11	-
Gender			
Male	92.2% (83)	63.6% (7)	0.112
Female	17.8% (16)	36.4% (4)	
Age groups			
≤55 years	56.7% (51)	63.6% (7)	0.534
>55 years	53.3% (48)	36.4% (4)	
Body mass index (kg/m^2^)			
≤25	62.2% (56)	63.6% (7)	0.756
>25	47.8% (43)	36.4% (4)	
Diabetes mellitus			
Yes	44.4% (40)	36.4% (4)	>0.999
No	65.6% (59)	63.6% (7)	
Hypertension			
Yes	74.4% (67)	63.6% (7)	0.748
No	35.6% (32)	36.4% (4)	
Smoking			
Yes	54.4% (49)	36.4% (4)	0.530
No	55.6% (50)	63.6% (7)	
Dyslipidemia			
Yes	35.6% (32)	27.3% (3)	>0.999
No	74.4% (67)	72.7% (8)	
Family history of coronary artery disease			
Yes	24.4% (22)	9.1% (1)	0.453
No	85.6% (77)	90.9% (10)	

## Discussion

This study was performed to evaluate the chances of MVD and subsequent TIMI flow grade in patients who have undergone primary PCI. We observed that half (50%) of the patients had MVD, and a TIMI flow grade of III was attained in 90% of the patients after primary PCI. Slow flow/no-reflow (TIMI flow grades 0-II) after primary PCI plays a substantial role in the eventual diagnosis of the patients. However, we have observed no significant association between post-procedure TIMI flow grade and the number of vessels involved.

Multivessel involvement in patients with STEMI is not an uncommon finding; 50% MVD in our study is aligned with the reported frequency of as high as 44% to 66% in patients with STEMI [10-12,14,15]. MVD has been proven to be a significant prognostic marker with significantly higher rates of associated morbidities and mortalities [10-12]. Multivessel involvement is found to be associated with various clinical and demographic factors such that patients with MVD are older in age as compared to patients with SVD, comorbid conditions such as HTN and DM are more common, and patients with MVD have more complex and severe diseases with a higher proportion of type C lesions and a higher thrombus grade [10,12,14,16,17]. In this study, MVD was found to be also associated with dyslipidemia, family history of premature CADs, and female gender.

The primary aim in the treatment of patients with STEMI is successful reperfusion therapy, which may be performed through physical or pharmacological intervention [18,19]. The goal of all treatments, whether physical or pharmacological, is to restore the blood flow within the vessels. PCI was attempted for the culprit artery only, and almost 90% of the subjects exhibited TIMI flow grade III after the procedure. Primary PCI is not successful in restoring myocardial perfusion in a significant number of patients. In our study, approximately 10% of subjects exhibited TIMI flow grade 0 to II after the procedure. This is referred to as the slow/no-reflow phenomenon and is often linked with negative outcomes following the procedure [20-24]. The exact mechanism behind the slow/no-reflow phenomenon is not clear; however, various clinical characteristics are associated with this phenomenon such as advanced age, delayed reperfusion, a longer length of the lesion, collateral flow, decreased left ventricular ejection fraction, MVD, thrombus grade, increased heart rate, elevated creatinine, and presence of comorbid conditions such as DM and HTN [20,23-26].

Therefore, both multivessel involvement and post-procedure slow/no-reflow have prognostic implications in patients with STEMI. Primary PCI in these patients is not only safe but also a key strategy for the early restoration of the myocardium when performed timely. For patients with MVD, culprit lesion-only PCI strategy is a common practice. However, multivessel PCI strategy can be adopted in certain cases. With its potential benefits, it may also possess certain clinical disadvantages such as hemodynamics of the patients may be compromised due to extended duration and increased use of contrast during the intervention of non-culprit lesion [10,27-30].

One of the limitations of our study is a relatively smaller sample size, but it was conducted at the largest cardiac center of the country, covering almost all the population subgroups. Another limiting factor of our study was the male-centric nature of our sample, with almost 81% being represented by men. Therefore, the data cannot be implicated to conclude gender-based differences in TIMI flow grade after primary PCI.

## Conclusions

Post-procedure TIMI flow grade III was accomplished in most subjects with or without MVD. Therefore, primary PCI plays a vital role in the early restoration of the blood flow to the myocardium during STEMI regardless of the number of vessels involved.
